# Impact of maximal exercise on immune cell mobilization and bioenergetics

**DOI:** 10.14814/phy2.15753

**Published:** 2023-06-13

**Authors:** James E. Stampley, Eunhan Cho, Haoyan Wang, Bailey Theall, Neil M. Johannsen, Guillaume Spielmann, Brian A. Irving

**Affiliations:** ^1^ School of Kinesiology Louisiana State University Baton Rouge Louisiana USA; ^2^ College of Physical Education and Health Sciences Zhejiang Normal University Jinhua China; ^3^ Pennington Biomedical Research Center Louisiana State University Baton Rouge Louisiana USA

**Keywords:** athletes, inflammation, mitochondria, oxygraph, PBMC, respirometry

## Abstract

Acute aerobic exercise increases the number and proportions of circulating peripheral blood mononuclear cells (PMBC) and can alter PBMC mitochondrial bioenergetics. In this study, we aimed to examine the impact of a maximal exercise bout on immune cell metabolism in collegiate swimmers. Eleven (7 M/4F) collegiate swimmers completed a maximal exercise test to measure anaerobic power and capacity. Pre‐ and postexercise PBMCs were isolated to measure the immune cell phenotypes and mitochondrial bioenergetics using flow cytometry and high‐resolution respirometry. The maximal exercise bout increased circulating levels of PBMCs, particularly in central memory (KLRG1+/CD57−) and senescent (KLRG1+/CD57+) CD8+ T cells, whether measured as a % of PMBCs or as absolute concentrations (all *p* < 0.05). At the *cellularlevel*, the routine oxygen flow (*I*O_2_ [pmol·s^−1^·10^6^ PBMCs^−1^]) increased following maximal exercise (*p* = 0.042); however, there were no effects of exercise on the *I*O_2_ measured under the LEAK, oxidative phosphorylation (OXPHOS), or electron transfer (ET) capacities. There were exercise‐induced increases in the *tissue‐level* oxygen flow (*I*O_2‐tissue_ [pmol·s^−1^·mL blood^−1^]) for all respiratory states (all *p* < 0.01), except for the LEAK state, after accounting for the mobilization of PBMCs. Future subtype‐specific studies are needed to characterize further maximal exercise's true impact on immune cell bioenergetics.

## 1 INTRODUCTION

Although regular aerobic exercise positively affects the immune system and reduces the risk of illness (Campbell & Turner, 2018), the underlying mechanisms by which regular aerobic exercise enhances the immune system remain incompletely understood. Exercise‐induced leukocytosis was first described over 100 years ago in a study that examined blood from participants of the 1901 Boston Marathon (Larrabee, 1902). Over the last couple of decades, exercise immunologists have quickly advanced our understanding of the impact of acute exercise bouts on the mobilization of peripheral blood mononuclear cells (PBMCs) (Spielmann et al., 2014).

Acute exercise clearly mobilizes immune cells into the peripheral blood compartment (Spielmann et al., 2014). However, the study of exercise‐induced mobilization of PBMCs is confounded by the differential mobilization of immune cell subtypes with unique effector functions. Indeed, while various immune cells are mobilized with exercise, including B cells (Turner et al., 2016), NK cells (Bigley et al., 2015), and monocytes (Steppich et al., 2000), T cells are arguably among the most exercise‐responsive immune cell subtypes. For example, moderate‐to‐vigorous intensity exercise preferentially mobilizes highly differentiated and senescent T cells (Spielmann et al., 2014), along with other tissue‐residing innate lymphoid cells (ILCs) (Hanson et al., 2020). The preferential mobilization of these immune cell subtypes is likely due to transient increases in catecholamines and hemodynamic shear stress, which collectively promote cellular demargination and tissue extravasation (Graff et al., 2018). Considering the high expression of beta‐2 adrenergic receptors on highly differentiated and senescent T cells, these transient physiological responses to exercise likely induce their mobilization to the peripheral blood compartment and affect their bioenergetic responses (Graff et al., 2018). Furthermore, highly differentiated and senescent T cells have been shown to have impaired metabolic flexibility and mitochondrial oxidative capacity (OXPHOS) while favoring glycolysis for their effector functions (Almeida et al., 2016; Geltink et al., 2018; Hortova‐Kohoutkova et al., 2021). Moreover, previous research has shown that circulating levels of epinephrine and norepinephrine are particularly high during maximal exercise in highly trained adults (Kjaer et al., 1985), which may accentuate the aforementioned responses.

Emerging evidence suggests that some of the beneficial effects that regular exercise has on the immune system are likely mediated by alterations in immune cell bioenergetics (Nieman & Pence, 2020; Rosa‐Neto et al., 2022). However, the data assessing the impact of acute exercise bouts on the bioenergetics of PBMCs and their respective immune cell subtypes are just beginning to emerge. For example, a recent study revealed that 60 min of low‐intensity (~35% VO_2 peak_) exercise was associated with increased routine respiration and fat oxidation measured in PBMCs acquired from sedentary adults unaccustomed to performing the acute bout of exercise, when normalized per million cells (Liepinsh et al., 2020). In contrast, we recently reported that 30 minutes of moderate‐intensity (~70% VO_2 peak_) exercise had no effect on routine respiration or measures of mitochondrial OXPHOS in PBMCs when normalized per million cells (e.g., pmol·s^−1^·10^6^ PBMCs^−1^, *cellular‐level* respiration) (Theall et al., 2021). However, after accounting for the exercise‐induced increase in PBMC concentrations, which leads to increased concentrations of PBMC‐associated mitochondria per milliliter of blood, we noted increased rates of routine respiration as well as measures of mitochondrial OXPHOS (e.g., pmol·s^−1^·mL blood^−1^, *tissue‐level* respiration) (Theall et al., 2021). This contrasts with mitochondrial OXPHOS data obtained from muscle tissues, where the number of mitochondria remains unchanged at the *tissue‐level* following an acute bout of exercise.

Considering the well‐documented metabolic (Hardin et al., 1995) and immune (Minuzzi et al., 2018; Zacher et al., 2022) adaptations to exercise training observed in athletes, it is crucial to assess whether the preferential mobilization of highly differentiated and senescent T cells in response to acute exercise that occurs in elite level athletes affects PBMC bioenergetics. This study aimed to determine the impact of a maximal exercise bout designed to measure anaerobic power and capacity on immune metabolism by measuring PBMC mitochondrial respiration determined using high‐resolution respirometry and immune cell phenotypes using flow cytometry in highly trained collegiate swimmers. We hypothesized that a maximal exercise bout would (i) increase PBMC mitochondrial respiratory function both at the *cellular‐* and *tissue‐levels* and (ii) lead to a preferential mobilization of highly differentiated and senescent T cells.

## 2 METHODS

### 2.1 Participants

As previously reported (Theall et al., 2020), 15 National Collegiate Athletic Association (NCAA) Division I swimmers were recruited and participated in a study designed to examine the impact of 7 months of collegiate swim training on exercise performance and immune cell responses to a maximal exercise bout (swimming) measured at multiple time points throughout the season. This analysis includes a subset of 11 (7 M/4F) of the participants who had complete pre‐ and postexercise high‐resolution respirometry data, which was assessed during either their preseason time point (*n* = 8) or postseason time point (*n* = 3), thus *n* = 11 independent study visits. The Louisiana State University Institutional Review Board provided ethics approval for this study (IRB#3836), and all participants provided written informed consent. The participants' heights and weights were measured during their initial screening visit (Table 1).

**TABLE 1:** Participant characteristics.

	Median	Interquartile range
Age, year	20	(20, 20)
Height, cm	180.3	(170.2, 182.9)
Weight, kg	79.1	(63.6, 81.7)

### 2.2 Maximal exercise (swimming) bout

After an overnight fast, the participants provided a pre‐exercise blood sample and then completed a 5‐min low‐intensity warm‐up swim. Next, the participants completed an in‐water maximal exercise test as previously described (Theall et al., 2020). In brief, the participants completed a series of all‐out 22.86 m (25 yard) swims with increasing resistance designed to measure anaerobic power and capacity (Theall et al., 2020). The participants wore a harness tethered to a pulley‐based, bucket system designed to allow for increases in resistance while swimming freely. Male participants performed their first all‐out swim trial with 18 kg (40 Ibs.) in the bucket, while female participants had 9 kg (20 lbs.) in the bucket. They were given three‐minute recovery periods between successive trials. After each successful trial, 9 kg (20 Ibs) or 7 kg (15 Ibs.) was added to the buckets of the male and female participants, respectively. The test was terminated when the participant could not complete the 22.86 m (25 yard) swim within a given trial. Participants were instructed to immediately exit the pool to provide a postexercise blood sample within 3 minutes upon test termination. Total work was calculated based on the total distance the bucket traveled during the test, accounting for increases in the weight of the bucket. The total distance traveled by the bucket was measured using a tape measure that was monitored using a high‐speed digital camera (GoPro Hero 4, San Mateo, Ca). Heart rates, blood lactate concentrations, and ratings of perceived exertion were collected at the end of each trial (Theall et al., 2020). Note: the maximal exercise bouts were performed ~24–48 hours after their last training session.

### 2.3 Blood collection and PBMC isolation

Blood was collected pre‐ and immediately postexercise by a trained phlebotomist using a butterfly needle and transferred to K_2_EDTA (BD vacutainer) tubes. As previously described, the samples were gently rocked at room temperature before the isolation of peripheral blood mononuclear cells (PBMCs) (Theall et al., 2020). Briefly, blood samples were diluted into equal parts PBS and layered over histopaque (Histopaque; Sigma‐Aldrich, St. Louis, MO) into 15‐mL centrifuge tube at room temperature. Samples were then centrifuged at 800*g* for 30 minutes and washed twice with PBS. Cells were counted via flow cytometry (BD Accuri, Ann Arbor, MI) and manually using a hematocytometer before antibody labeling. Once the samples were counted, they were divided into separate tubes containing four to five million cells for either phenotyping via flow cytometry or mitochondrial respiration measurement using high‐resolution respirometry.

### 2.4 Flow cytometry

We assessed T cell phenotypes using four‐color flow cytometry on a BD Accuri C6 flow cytometer as previously described (Theall et al., 2020,2021). In brief, isolated pan T cells (1.0 × 10^6^) were labeled with prediluted monoclonal antibodies (mAbs) and incubated at room temperature in the dark for 30 min. The mAb combinations consisted of antikiller cell lectin‐like receptor G1 (KLRG1) Alexa488, CD57 PE, CD4 PerCP or CD8 PerCP, and CD3 APC to allow for comprehensive characterization of the level of T cell differentiation.

### 2.5 High‐Resolution respirometry

We used high‐resolution respirometry to measure *cellular‐level* oxygen flow (*I*O_2_, pmols∙s^−1^∙10^6^ PBMCs^−1^) in isolated PBMCs using a standardized substrate‐uncoupler‐inhibitor (SUIT) protocol. Specifically, following the isolation of the PBMCs, they were washed in PBS, counted using a flow cytometer, and subsequently resuspended in mitochondrial respiration buffer (MiR05, 0.5 mM EGTA, 3 mM MgCl_2_, 60 mM Lactobionic Acid, 20 mM Taurine, 10 mM KH_2_PO_4_, 20 mM HEPES, 110 mM Sucrose, and 1 g/L Fatty Acid Free BSA, pH 7.1) as described above. Approximately 4 million pre‐exercise PBMCs were added to chamber A, and 4 million postexercise PBMCs were added to chamber B (or vice versa) of commercially available high‐resolution respirometer (Oxygraph O2k, Oroboros). Each chamber contained 2 mL of MiR05, and all measurements were acquired at 37°C, with oxygen concentrations between ~200 and 50 μM. Before adding the samples, we performed a daily room air calibration per the manufactures' instructions. We measured routine *I*O_2_ in unpermeabilized PBMCs in the absence of exogenous substrates (CE). We then titrated in digitonin (~8 μg/million PBMCs) to chemically permeabilize the plasma membranes of the PBMCs. Next, in the presence of pyruvate (5 mM) and malate (2 mM) and the absence of ADP, we measured NADH‐linked LEAK (N_*L*_) *I*O_2_. We then measured NADH‐linked oxidative (OXPHOS) capacity (N_*P*_) *I*O_2_ after adding saturating levels of ADP‐Mg^++^ (2.5 mM). We then added cytochrome C (20 μM) to measure the integrity of the mitochondrial membrane. We then titrated in glutamate (10 mM) to further assess the N_*P*_ *I*O_2_. Next, we titrated succinate (10 mM) to measure NADH&Succinate‐linked OXPHOS (NS_*P*_) *I*O_2_. We then titrated additional ADP (5 mM) to determine whether the ADP‐Mg^++^ concentration was still saturating. Note: All OXPHOS measurements were measured with 2.5 mM ADP, which was sufficient to saturate both the N_*P*_ and NS_*P*_ pathways. To measure NADH&Succinate‐linked electron transfer (ET) capacity (NS_E_) *I*O_2,_ we titrated in carbonyl cyanide m‐chlorophenyl hydrazine (CCCP) (0.15–0.5 μM/steps). Finally, we added 2.5 μM antimycin A to measure residual O_**2**_ consumption (R_*OX*_) *I*O_2_. In addition to normalizing the *I*O_2_ at the *cellular‐level*, we also normalized the *I*O_2_ to *tissue‐level* (*I*O_*2‐Tissue*_, pmols∙s^−1^∙mL blood^−1^) by multiplying the *I*O_2_ by the PBMCs concentration in the whole blood, which was measured using a clinical‐grade hematology analyzer (Beckman Coulter, CA, USA). In addition, we also calculated the flux control ratios (FCRs) by dividing the *I*O_2_ measurements acquired during the individual respiratory states by the *I*O_2_ during the NS_*E*_ state. Three participants from the original cohort who provided pre‐ and postexercise blood samples were excluded from this analysis due to poor sample quality in one of their samples and one participate did not have a pre‐ or postexercise sample for this analysis.

### 2.6 Statistical analysis

Data were analyzed using *JMP16.2* (SAS Institute). We present data as their median and interquartile range (25th, 75th %ile) unless otherwise noted. We used Wilcoxon matched pairs signed‐rank test to determine whether the pre‐ vs. postexercise measurements were statistically different at *α* = 0.05. We used nonparametric tests due to the small sample size and non‐normally distributed nature of the data.

## 3 RESULTS

### 3.1 Maximal exercise performance

All 11 participants in this analysis completed the maximal exercise (swimming) bout and provided pre‐ and postexercise blood samples. The median (interquartile range) work completed during the bucket test was 2170 (1176–2658) Joules (*n* = 10). Of note, one participant completed the bucket test, but the total work performed could not be accurately calculated due to a camera malfunction. Measured at the completion of the last trial, the heart rate was 166 (155–170) beats per min, the blood lactate concentration was 10.5 (10.3–12.7) mM, and the RPE was interquartile range (19–20) (*n* = 11). Moreover, after the last trial, the heart rate as a percentage of age‐predicted (220‐age) max was 83% (77.5%–84.6%).

### 3.2 Maximal exercise‐induced changes in T‐Cell phenotype

**FIGURE 1:**
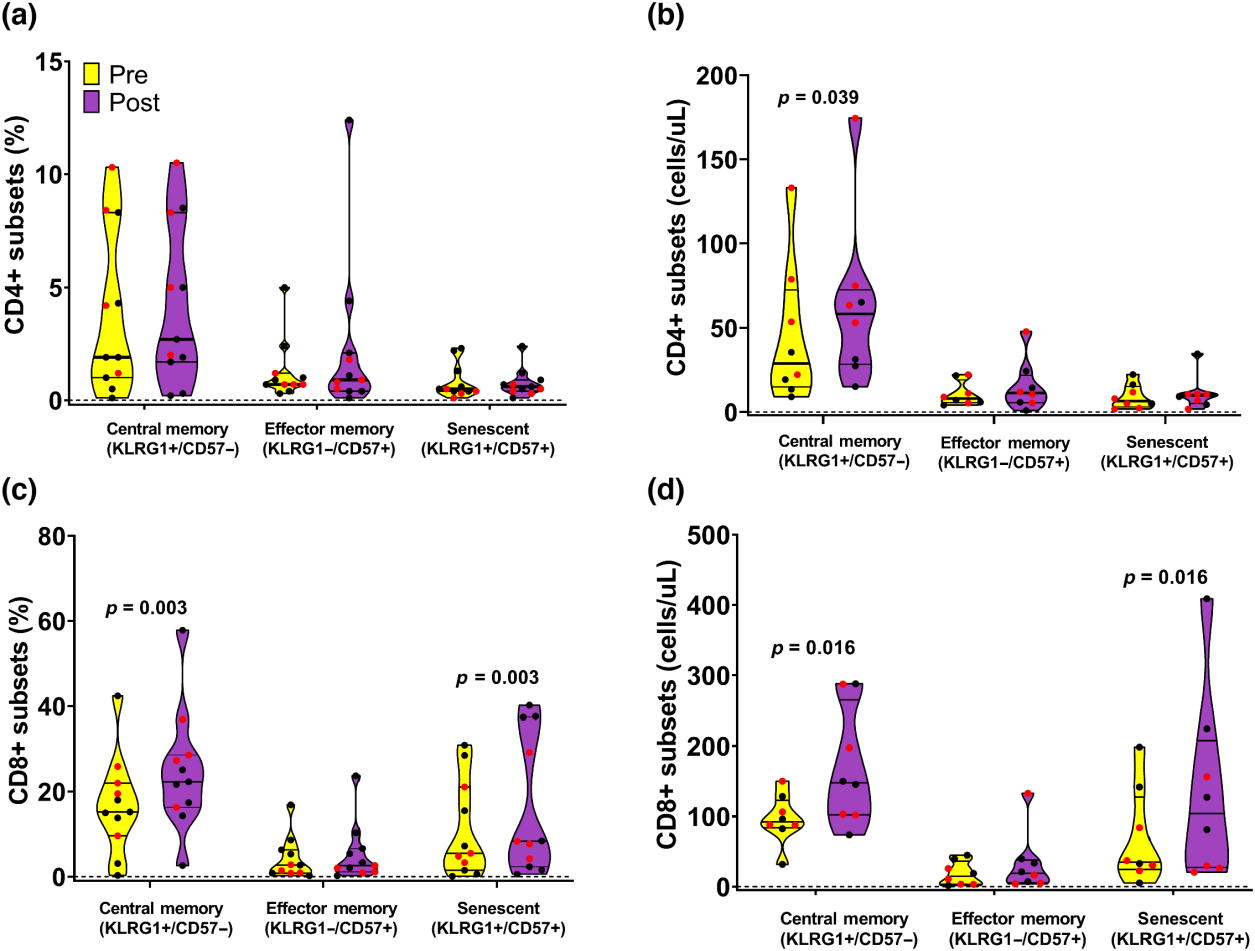
Panel (a) presents truncated violin plots of frequencies of central memory, effector memory, and senescent CD4^+^ T cells measured in pre‐ (yellow) and postexercise (purple) blood samples acquired from the collegiate swimmers following a maximal exercise bout. Panel (b) presents truncated violin plots of concentration (cells/uL) of central memory, effector memory, and senescent CD4^+^ T cells measured in pre‐ (yellow) and postexercise (purple) blood samples from the collegiate swimmers following a maximal exercise bout. Panel (c) presents truncated violin plots of frequencies of central memory, effector memory, and senescent CD8^+^ T cells measured in pre‐ (yellow) and postexercise (purple) blood samples acquired from the collegiate swimmers following a maximal exercise bout. Panel (d) presents truncated violin plots of concentration (cells/uL) of central memory, effector memory, and senescent CD8^+^ T cells measured in pre‐ (yellow) and postexercise (purple) blood samples from the collegiate swimmers following a maximal exercise bout. The truncated violin plots show the minimum, 25th, 50th, 75%ile, and maximum. The black (male) and red (females) circles represent each participant's data. Wilcoxon matched pairs singed‐rank tests were used to test for significant pre‐ to postexercise differences within each T cell subtype.

**FIGURE 2:**
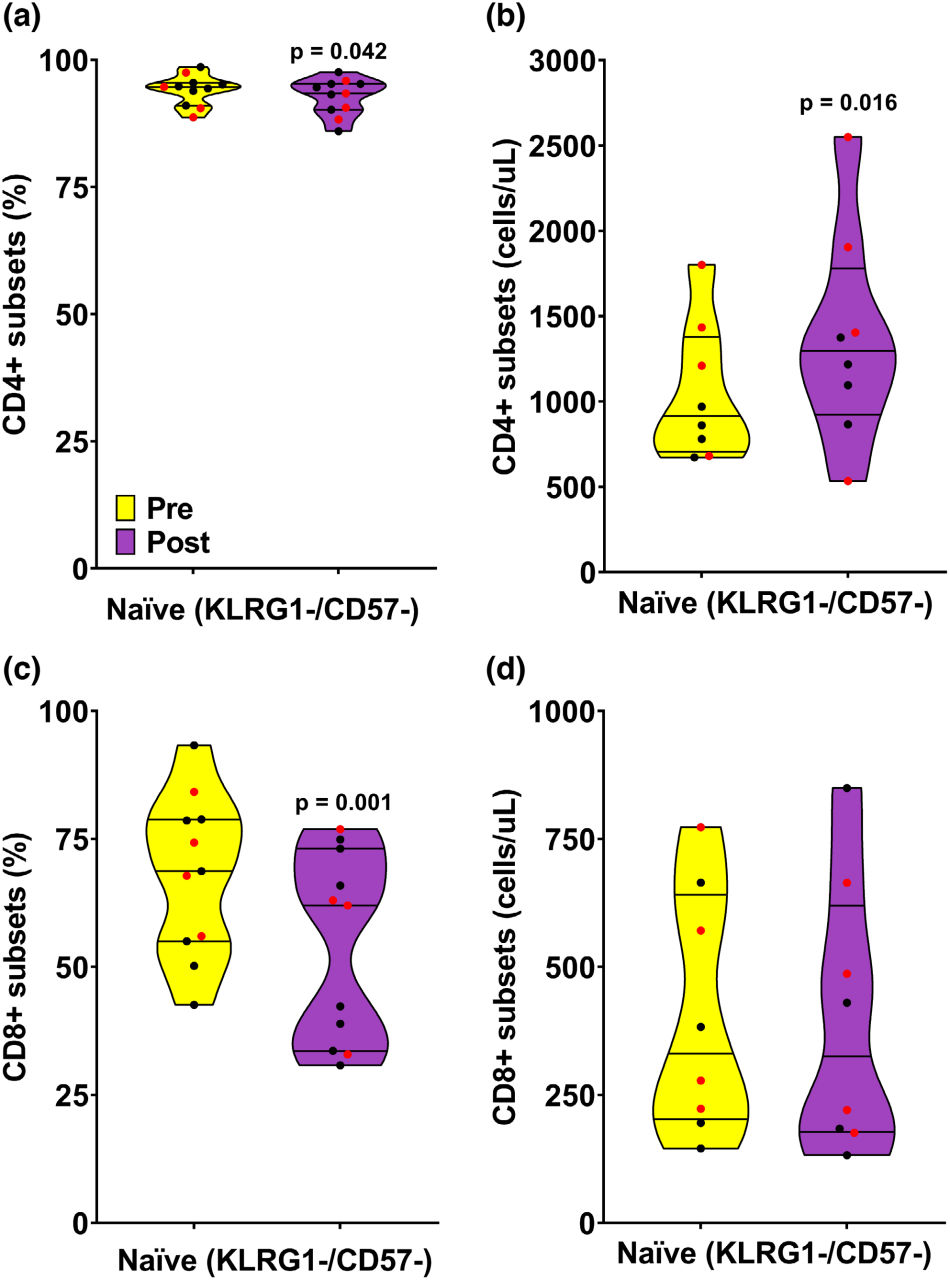
Panel (a) presents truncated violin plots of frequencies of naïve CD4^+^ T cells measured in pre‐ (yellow) and postexercise (purple) blood samples acquired from the collegiate swimmers following a maximal exercise bout. Panel (b) presents truncated violin plots of concentration (cells/uL) of naïve CD4^+^ T cells measured in pre‐ (yellow) and postexercise (purple) blood samples from the collegiate swimmers following a maximal exercise bout. Panel (c) presents truncated violin plots of frequencies of naïve CD8^+^ T cells measured in pre‐ (yellow) and postexercise (purple) blood samples acquired from the collegiate swimmers following a maximal exercise bout. Panel (d) presents truncated violin plots of concentration (cells/uL) of naïve CD8^+^ T cells measured in pre‐ (yellow) and postexercise (purple) blood samples from the collegiate swimmers following a maximal exercise bout. The truncated violin plots show the minimum, 25th, 50th, 75%ile, and maximum. The black (male) and red (females) circles represent each participant's data. Wilcoxon matched pairs singed‐rank tests were used to test for significant pre‐ to postexercise differences within each T cell subtype.

Figure 1 presents the impact of the maximal exercise bout on the frequencies of circulating CD4+ and CD8+ T cells, specifically focusing on the central memory, effector memory, and senescent cell subtypes. Figure 2 presents the impact of the maximal exercise bout on CD4+ and CD8+ naïve cell T cells. The maximal exercise bout did not affect the frequency of circulating CD4+ KLRG1+/CD57‐ central memory, KLRG1‐/CD57+ effector memory, or KLRG1+/CD57+ senescent T cells (Figure 1a). Moreover, the numbers of CD4+ KLRG1+/CD57‐ central memory T cells increased following the maximal exercise bout (Figure 1b). However, the frequency of naïve KLRG1‐/CD57‐ CD4+ T cells was lower postexercise than pre‐exercise (Figure 2a), while their overall numbers increased (Figure 2b). Concerning the CD8+ T cells, both the frequencies and numbers of circulating KLRG1+/CD57‐ central memory and KLRG1+/CD57+ senescent T cells increased following the maximal exercise bout (Figure 1c,d). Finally, the frequency of circulating KLRG1‐/CD57‐ naïve CD8+ T cells decreased following the maximal exercise bout (Figure 2c); however, this did not translate to a change in the overall numbers of naïve KLRG1‐/CD57‐ CD8+ T cells (Figure 2d).

### 3.3 Maximal exercise‐induced changes in PBMC bioenergetics

**FIGURE 3:**
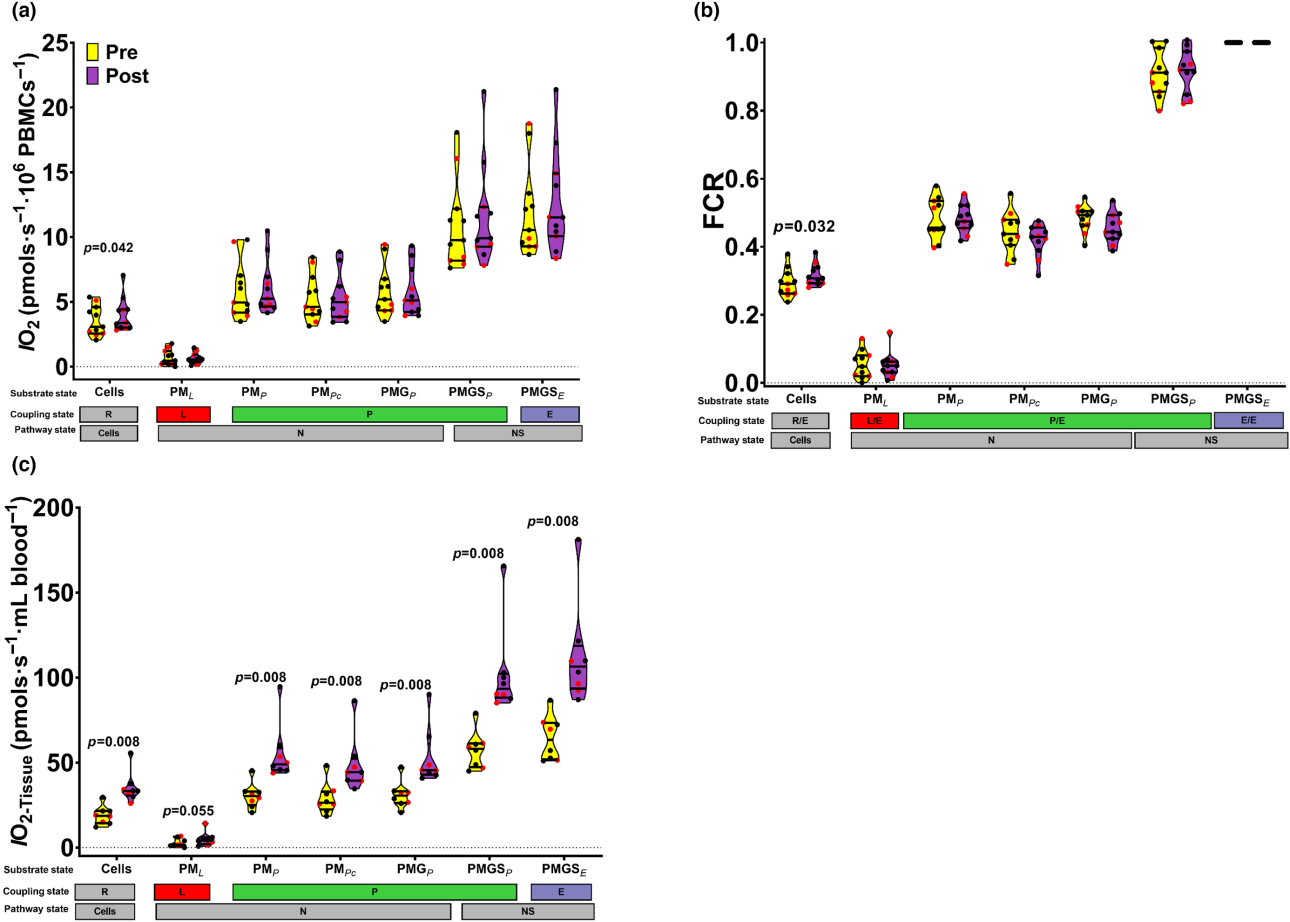
Panel (a) presents truncated violin plots of the *cellular‐level* oxygen flow (*I*O_2_ [pmol·s^−1^·10^6^ PBMCs^−1^]) measured in intact and digitonin permeabilized PBMCs acquired pre‐ (yellow) and immediately postexercise (purple) from 11 collegiate swimmers following a maximal exercise bout. The *I*O_2_ was measured under routine (R), LEAK (L), oxidative capacity (P), and ET capacity (E) respiratory control states, under the following substrate conditions endogenous substrates (cells), NADH‐linked substrates (N), and NADH&Succinate‐linked (NS) substrates. The raw *I*O_2_ were corrected for the residual oxygen consumption measured following the addition of 2.5 μM antimycin A. Panel (b) presents truncated violin plots of the respective flux control ratios (FCR), the FCR were calculated by normalizing the *I*O_2_ under the different respiratory control states by the *I*O_2_ under the E state with NS‐linked substrates). Panel C presents truncated violin plots of the *tissue‐level* oxygen flow (*I*O_2‐Tissue_[pmol·s^−1^·mL blood^−1^]) of the respective respiratory control states and substrate conditions. The *I*O_2‐Tissue_ was calculated by multiplying the *I*O_2_ by the PBMC concentration in the whole blood. Measurements were taken using high‐resolution respirometry at 37°C with O_2_ concentrations between ~200 μM and ~ 50 μM in MiR05. PM_L_: (5 mM pyruvate, 2 mM Malate, no ADP), PM_P_: (PM + 2.5 mM ADP‐Mg^++^), PM_Pc_ (PM_P_ + 20 μM cytochrome c), PMG_P_ (PM_Pc_ + 10 mM glutamate), PMGS_P_ (PMG_P_ + 10 mM succinate), PMGS_E_ (PMGS_P_ + 0.15–0.5 μM/steps of carbonyl cyanide m‐chlorophenyl hydrazine (CCCP)). The truncated violin plots show the minimum, 25th, 50th, 75%ile, and maximum. The black (male) and red (females) circles represent each participant's data. Wilcoxon matched pairs singed‐rank tests were used to test for significant pre‐ to postexercise differences.

Figure 3a presents the pre‐ and postexercise *cellular‐level* oxygen flow (*I*O_2_ [pmol·s^−1^·10^6^ PBMCs^−1^]) measured in intact and digitonin permeabilized PBMCs. At the *cellular‐level*, the routine intact *I*O_2_ was increased following the maximal exercise bout (*p* = 0.042), while the *I*O_2_ during all the other respiratory states were unaffected by the maximal exercise bout. Likewise, the FCR for routine respiration was also increased following the maximal exercise bout (*p* = 0.032), while the FCRs under all the other respiratory states were unaffected by the maximal exercise bout (Figure 3b).

Since the maximal exercise bout significantly increased the concentration of PBMCs in the blood, we also assessed the impact of the maximal exercise bout on the *tissue‐level* oxygen flow (*I*O_2‐Tissue_[pmol·s^−1^·mL blood^−1^]). After accounting for the exercise‐induced increase in PBMC concentrations in the blood, there were increases in the *I*O_2‐Tissue_ for all respiratory states following the maximal exercise bout (all *p* < 0.01), except for the LEAK state (Figure 3c).

## 4 DISCUSSION

The objective of this study was to investigate the impact of a maximal exercise bout (i.e., the bucket test) on PBMC bioenergetics by measuring mitochondrial oxygen flow (respiration) both at the *cellular‐level* (*I*O_2_[pmol·s^−1^·10^6^ PBMCs^−1^]) and *tissue‐level* (*IO*_*2‐Tissue*_[pmols∙s^−1^∙mL blood^−1^]) after accounting for the well‐documented exercise‐induced immune cell redistribution in the peripheral blood compartment (Theall et al., 2021). The primary findings of the present study were that although a maximal exercise bout led to minimal changes in unstimulated *cellular‐level I*O_2_, it induced consistent elevations in *tissue‐level IO*_*2‐Tissue*_ in our collegiate swimmers. These results suggest that the maximal exercise bout's impact on unstimulated PBMC bioenergetics is likely mediated by exercise‐induced immune cell mobilization and redistribution rather than changes at the cellular level.

Since the *cellular‐level I*O_2_ has been shown to be elevated in physically fit vs. unfit adults (Janssen et al., 2022) and following acute endurance‐based exercise bouts in some (Liepinsh et al., 2020), but not all studies (Gatterer et al., 2018; Theall et al., 2021), additional studies were warranted. In the present study, the maximal exercise bout designed to measure anaerobic power and capacity increased *cellular‐level I*O_2_ by ~13% under the routine (basal) respiratory condition. However, the maximal exercise bout did not affect the *cellular‐level I*O_2_ under the LEAK, OXPHOS, and ET capacity states. As such, the Flux Control Ratio (FCR) under the routine respiratory condition was also higher postexercise than pre‐exercise. The present study is consistent with our prior study (Theall et al., 2021), in sedentary adults, which showed that 30 min of moderate‐to‐vigorous intensity (~70% VO_2 peak_) cycle ergometry did not affect *cellular‐level I*O_2_ under the LEAK, OXPHOS, or ET capacity states of mixed PBMC. The slight elevation in *cellular‐level I*O_2_ under the routine (basal) condition could be potentially driven by elevations in circulating levels of epinephrine and norepinephrine that can be achieved when performing maximal exercise, particularly, in highly trained adults (Kjaer et al., 1985). Moreover, highly differentiated T cells express a greater amount of beta‐2 adrenergic receptors (Fan & Wang, 2009; Graff et al., 2018; Slota et al., 2015), potentially making them more responsive to exercise‐induced epinephrine and norepinephrine stimulation. Unfortunately, we did not have sufficient sample volume to measure the circulating levels of these catecholamines. In contrast, 1 hour of low‐intensity (~35% VO_2 peak_) cycling has been shown to increase *cellular‐level I*O_2_ under routine, LEAK and OXPHOS capacity states using fatty acid‐derived substrates in sedentary adults (Liepinsh et al., 2020). In comparison, both of the studies by our group, including the present one, used carbohydrate‐based substrates. Interestingly, recent data also suggest that resistance‐based exercise that include both eccentric and concentric muscle contractions leads to reduced *cellular‐level I*O_2._ Collectively, these studies indicate that acute exercise bouts' impact on PBMC *cellular‐level I*O_2_ is likely dependent on the type of exercise (e.g., endurance, resistance, and anaerobic), duration of exercise, and training status of the participants. Moreover, the impact that the types of substrates used (carbohydrate vs. fatty acid) to interrogate PBMC mitochondrial respiratory function requires further study.

At *tissue‐level*, we observed that the postexercise samples had significantly higher *IO*_*2‐Tissue*_ than the pre‐exercise samples across all respiratory states after accounting for the exercise‐induced mobilization of immune cells into the peripheral blood compartment. The exercise‐induced increase in *IO*_*2‐Tissue*_ is consistent with our prior study in sedentary adults (Theall et al., 2021). Thus, we postulate that the biggest impact of acute exercise on PBMC mitochondrial bioenergetics is primarily mediated through the mobilization of immune cells into the peripheral blood compartment. To further understand the impact that acute exercise bouts have on PBMC bioenergetics, we also considered the preferential mobilization of immune cell subtypes (e.g., CD4+ T‐cells vs. CD8+ T‐cells). When examining the acute effect of maximal exercise on phenotype‐specific mobilization, we see that postexercise CD4+ and CD8+ T cells have higher percentages and concentrations of central memory cells when compared to pre‐exercise. However, the exercise‐induced increase in CD4+ T cells did not reach the level of statistical significance. Of interest, the maximal exercise bout increased the percentage and concentration of senescent CD8+ T cells. In addition, we also observed an exercise‐induced reduction in the percentage of Naïve CD8+ T cells. The preferential mobilization of central memory and senescent CD8+ T cells, with a concomitant reduction in Naïve CD8+ T cells as indicated by their overall percentages in the PBMC pool, likely has important effects on the *cellular‐level I*O_2._ For example, Naïve CD8+ T cells rely heavily on their ability to oxidize glucose (Corrado & Pearce, 2022; Rangel Rivera et al., 2021). Compared with Naïve CD8+ T cells, central memory T cells are thought to rely on a mixture of OXPHOS, fatty acid oxidation, and glycolysis (Corrado & Pearce, 2022; Rangel Rivera et al., 2021), while senescent CD8+ T cells relying on a mixture of OXPHOS and glycolysis as the begin to lose their metabolic flexibility (Corrado & Pearce, 2022; Rangel Rivera et al., 2021). Taken together, the exercise‐induced elevations in central memory and senescent CD8+ T cells percentages could potentially offset the slight decline in Naïve CD8+ T cells percentages with respect to exercise induced changes in *cellular‐level I*O_2_ for the overall PBMC pool_._

Since PBMC samples are essentially a mixture of different cell types (e.g., lymphocytes, monocytes, and B cells) and subtypes (e.g., CD4+ vs. CD8+ T cells), interpreting any potential impact that acute exercise has on changes to bioenergetic output becomes challenging as noted above. Thus, future studies are needed to comprehensively characterize the impact of acute exercise on the bioenergetics of purified immune cell subtypes, which would further support recent data on the importance of understanding mitochondrial phenotypes in purified immune cell subtypes and mixtures (Rausser et al., 2021). For example, isolating specific immune cell subtypes would likely provide a more focused picture of how acute exercise alters mitochondrial respiratory function in defined immune cell subtypes. Since CD8+ T cells and NK cells have been shown to have some of the highest levels of mobilization (Bigley et al., 2014; Campbell et al., 2009), characterizing their mitochondrial function in response to exercise should be a priority of future research. Moreover, emerging data also suggests that the mitochondrial phenotypes in multiple immune cell subtypes are affected by sex. Thus, studies that are sufficiently powered to assess the impact of sex on exercise‐induced changes in immune cell bioenergetics are needed. In addition, it should be noted that this study only assessed the mitochondrial function of unstimulated PBMCs. Considering the documented link between bioenergetic and effector functions, future studies should characterize the mitochondrial function of activated postexercise immune cells to increase the clinical relevance of these findings. Another factor to consider is the physical fitness status of the participants. The present study aimed to assess the effects of a maximal exercise bout on PBMC mitochondrial function in highly trained collegiate athletes. However, considering that physical inactivity and prolonged sedentary behaviors are known to detrimentally impact immune competency and PBMC composition (Spielmann et al., 2011), it is likely that changes in PBMC mitochondrial function in response to acute exercise would be different in unfit individuals. Similarly, older adults are known to have a greater proportion of circulating senescent T cells than their younger counterparts, especially when sedentary (Duggal et al., 2018; Spielmann et al., 2011). The current study focused on young adults with high level of physical fitness, which at least partially explains the lack of difference in PBMC respiration in response to acute exercise at the cellular level. It is likely that age and sedentary behavior would only exacerbate the effects of exercise on PBMC mitochondrial function. For this reason, another potential direction for future studies would be to explore the different responses to exercise from different populations based on their current level of physical activity and age.

## 5 CONCLUSION

In conclusion, for the first time in collegiate swimmers, we demonstrated that a maximal exercise bout (i.e., the bucket test) was associated with minimal changes in *cellular‐level I*O_2._ In contrast, due to the robust mobilization of immune cells in response to the maximal exercise bout, the *tissue‐level IO*_*2‐Tissue*_ was significantly elevated. Moreover, the preferential mobilization of central memory and senescent T cells with a concomitant reduction in Naïve T cells could have masked important *cellular‐level* changes in mitochondrial respiratory function when measured at the PBMC level, which is a mixture of metabolically distinct immune cell subtypes. Future subtype‐specific studies are needed to provide a more comprehensive picture of the true impact of acute exercise on immune cell bioenergetics.

## AUTHOR CONTRIBUTIONS

Brian A. Irving, Guillaume Spielmann, and Neil M. Johannsen were involved in conceptualization and methodology; James E. Stampley, Eunhan Cho, Haoyan Wang, Bailey Theall, Neil M. Johannsen, Guillaume Spielmann, and Brian A. Irving were involved in data collection and analysis; James E. Stampley was involved in writing—original draft preparation; all authors were involved in writing—review and editing. All authors have read and agreed to the published version of the manuscript.

## CONFLICT OF INTEREST STATEMENT

The authors have no conflicts of interest to report.

## Data Availability

Data are available upon request.
